# Post-Discharge Trajectories of Romanian Premature Infants: A Cross-Sectional Analysis of Associated Factors

**DOI:** 10.3390/children12091107

**Published:** 2025-08-22

**Authors:** Ioana Rosca, Alexandru Dinulescu, Ana Prejmereanu, Mirela-Luminita Pavelescu, Alexandru Cosmin Palcău, Daniela-Eugenia Popescu, Andreea Teodora Constantin

**Affiliations:** 1 Department of Neonatology, Faculty of Midwifery and Nursery, “Carol Davila” University of Medicine and Pharmacy, 050474 Bucharest, Romania; ioana.rosca@umfcd.ro; 2 Department of Obstetrics and Gynecology, Clinical Hospital of Obstetrics and Gynecology “Prof. Dr. Panait Sârbu”, 3–5 Giulesti Street, 060251 Bucharest, Romania; 3 Department of Pediatrics, Faculty of Medicine, “Carol Davila” University of Medicine and Pharmacy, 050474 Bucharest, Romania; alexandru.dinulescu@drd.umfcd.ro (A.D.); ana.prejmereanu@rez.umfcd.ro (A.P.); 4 Department of Pediatrics, Emergency Hospital for Children “Grigore Alexandrescu”, 011743 Bucharest, Romania; 5 Department of General Surgery, Faculty of Medicine, “Carol Davila” University of Medicine and Pharmacy, 050474 Bucharest, Romania; alexandru-cosmin.palcau@drd.umfcd.ro; 6 Department of General Surgery, University Emergency Hospital of Bucharest, 050098 Bucharest, Romania; 7 Department of Obstetrics and Gynecology, “Victor Babeş” University of Medicine and Pharmacy, Eftimie Murgu Sq. No. 2, 300041 Timisoara, Romania; popescu.daniela@umft.ro; 8 Department of Neonatology, Premiere Hospital, Regina Maria Health Network, Calea Aradului, No. 113, 300645 Timisoara, Romania; 9 Pediatrics Department, National Institute for Mother and Child Health “Alessandrescu-Rusescu”, 020395 Bucharest, Romania; andreea.constatin@drd.umfcd.ro

**Keywords:** prematurity, neonatal follow-up, RSV prophylaxis, neonatal outcomes, Romania

## Abstract

(1) Background: Prematurity remains a leading contributor to neonatal morbidity and mortality, with long-term consequences that extend well beyond the neonatal period. This cross-sectional study aimed to identify key maternal, perinatal, and neonatal factors that influence the short- and long-term evolution of preterm infants. It also seeks to evaluate the level of post-discharge follow-up care, parental involvement during NICU hospitalization, access to supportive therapies such as physiotherapy and RSV prophylaxis, and the impact of breastfeeding practices. Additionally, the study explores parental awareness and use of recent national initiatives in Romania, such as the “Premature and Vulnerable Newborn’s Notebook”. (2) Methods: A total of 360 mothers of preterm infants born between 2001 and 2025 responded to a structured questionnaire assessing clinical characteristics, NICU care, parental involvement, and post-discharge support. (3) Results: The findings indicate that assisted reproduction and pregnancy plurality were associated with higher maternal age, while vaginal delivery was associated with lower gestational age at birth. Notably, only 25% of infants were enrolled in structured follow-up programs, and a large proportion of families relied on private services for physical therapy. Kangaroo mother care was reported by just 16.4% of mothers. While breastfeeding rates improved after discharge, access to multidisciplinary follow-up and public physiotherapy remains limited. Encouragingly, most mothers endorsed the proposed national initiative for a “Premature and Vulnerable Newborn’s Notebook.” (4) Conclusions: This study underscores the urgent need for a comprehensive national follow-up strategy to ensure equitable and continuous care for Romania’s vulnerable preterm population.

## 1. Introduction

Prematurity remains a leading contributor to neonatal morbidity and mortality, especially among extremely preterm infants, defined as those born before 28 weeks of gestation [1,2,3]. The incidence and severity of complications increase inversely with gestational age, resulting in a higher burden of mortality and long-term health issues among preterm neonates when compared to their term-born peers [4]. These survivors are at significant risk of developing chronic medical conditions and neurodevelopmental impairments, which often necessitate long-term medical, educational, and social support, thereby increasing the overall societal cost of care [5,6,7,8,9].

Recent qualitative research has underscored the vital role of family perceptions in shaping early intervention and neurodevelopmental care for preterm infants. In a study by Silva et al. [10], families reported a limited understanding of prematurity and neurodevelopmental risks, along with feelings of insecurity, particularly in the neonatal intensive care unit (NICU) environment and a tendency to perceive kangaroo care and post-discharge support as insufficient to foster independent caregiving. These findings point to significant gaps in parental awareness, which may impede effective engagement in developmental follow-up; therefore, understanding and addressing family perspectives should be a key component of improving access to supportive therapies and enhancing the overall continuity of care for preterm infants [10].

Despite major advancements in neonatal intensive care, which have led to improved survival rates among critically ill term and preterm infants, these newborns remain medically vulnerable and often require extended and comprehensive care beyond their (NICU) stay [11,12,13]. Studies have consistently shown that preterm infants, particularly those born at lower gestational ages, are more likely to experience recurrent hospitalizations during early childhood [14,15,16]. The neurodevelopmental outcome in preterm infants is influenced by many factors, including gestational age, birth weight, pregnancy or neonatal complications, and extended hospitalization [17]. 

Breastfeeding, and particularly feeding with human milk, offers substantial health benefits and is considered the optimal nutritional choice for all infants, especially for those born prematurely [18,19]. Human milk has demonstrated protective effects against serious neonatal complications such as necrotizing enterocolitis and late-onset sepsis; therefore, supporting and educating families on breastfeeding, beginning antenatally and continuing during NICU hospitalization, is essential [20]. Many NICUs now employ feeding readiness cues and support early oral feeding at the breast, even starting as early as 31 to 33 weeks corrected gestational age when suckling patterns become sufficiently coordinated [21,22,23].

Respiratory syncytial virus (RSV) poses a significant threat to preterm infants, particularly those with bronchopulmonary dysplasia (BPD), prompting the need for targeted preventive measures, such as palivizumab prophylaxis [24]. In 2023, the European Commission approved a maternal RSV vaccine (RSVpreF/Abrysvo^®^), allowing passive immunization through placental antibody transfer, with potential to significantly reduce infant readmissions [25,26].

In Romania, efforts to improve outcomes for preterm infants include the classification of maternity hospitals into three levels of care, with level 3 representing the highest level of neonatal complexity and support. Newborns delivered in level 1 maternities must be stabilized and transferred to at least a level 2 facility if the medical case exceeds their available resources, particularly in the case of prematurity [27]. The Romanian Chamber of Deputies has an initiative to introduce the “Premature and Vulnerable Newborn’s Notebook”, designed to support families by providing structured information about follow-up and required interventions [28]. There are also ongoing legislative efforts to extend parental leave to three years for mothers of preterm infants [29]. The follow-up for high-risk neonates in Romania is recommended in level 3 maternities, up to the age of 2 years, but the adherence to the follow-up is unknown. Bivoleanu et al. (2017) described a 72.7% adherence rate in 1157 preterm from Iasi County, Romania [30].

This study aims to identify key maternal, perinatal, and neonatal factors that influence the short- and long-term evolution of preterm infants. It also seeks to evaluate the level of post-discharge follow-up care, parental involvement during NICU hospitalization, access to supportive therapies such as physiotherapy and RSV prophylaxis, and the impact of breastfeeding practices. Additionally, the study explores parental awareness and use of recent national initiatives in Romania, such as the “Premature and Vulnerable Newborn’s Notebook”. Despite international attention to long-term outcomes among preterm infants, data on post-discharge follow-up, parental awareness, and access to supportive therapies remain scant in Romania. National monitoring systems and structured follow-up programs are unevenly implemented, and little is known about family experiences with early intervention, physiotherapy access, and uptake of measures such as RSV prophylaxis. Moreover, existing studies often rely on clinical registries and do not capture parental perceptions or private sector use of rehabilitation services. This study therefore addresses an important gap by characterizing (1) the extent and duration of structured follow-up after discharge; (2) parental contact and caregiving practices in the NICU and at home; (3) access to supportive therapies and prophylaxis—information necessary to inform national planning for developmental surveillance and family-centered interventions.

## 2. Materials and Methods

### 2.1. Study Setting

This is a cross-sectional retrospective study. A google forms questionnaire was sent to the mothers that had preterm newborns, and included questions about the characteristics of the mothers, pregnancy, newborns, and follow-up after discharge from maternity. The questionnaire consisting of 32 questions was distributed online on platforms such as Facebook, Instagram, and to different groups on WhatsApp. Online communities for mothers and the mothers from hospital records were targeted from the urban and well as rural regions in Romania.

We had answers from 360 women that gave birth in the interval of 1 January 2021–31 December 2024.

A structured questionnaire was developed by the research team to collect maternal, perinatal, and neonatal information, as well as post-discharge follow-up and parental perceptions. The instrument consisted of nine sections covering the domains listed in Table 1. To ensure alignment with the study aims, the questionnaire was structured into nine sections that mirror the objectives: maternal characteristics and method of conception (to identify prenatal risk factors), pregnancy and delivery details (to evaluate perinatal contributors), neonatal clinical data and NICU care (to capture in-hospital clinical course), parental contact and kangaroo mother care (to assess family involvement), feeding practices (to examine breastfeeding impact), enrollment and duration of structured follow-up and awareness of specialized centers (to quantify post-discharge surveillance), access to supportive therapies including physiotherapy and RSV prophylaxis (to evaluate service availability and private vs. public use), and infant health outcomes within the first year (to provide short-term outcome data). Each set of items was mapped a priori to specific aims and planned analyses—for example, questions on follow-up enrollment and duration measured the primary outcome of follow-up coverage, whereas questions on NICU contact and KMC informed secondary analyses of parental involvement and early feeding. The full questionnaire, including all items and response options, is provided in Appendix A.

Completion time was approximately 15–20 min. The questionnaire can be accessed at “https://forms.gle/k3yRGJnHasnbMmi28/” (accessed on 11 August 2025). The questionnaire was developed by the multidisciplinary study team (neonatology, pediatrics, and rehabilitation specialists) to capture domains relevant to post-discharge follow-up and parental experience. Although the instrument was not formally validated, items were drawn from previously published surveys and clinical practice guidelines and were reviewed for face validity by the research team. We acknowledge the lack of formal psychometric validation as a limitation.

For the purposes of this study, short-term evolution referred to the clinical course and health outcomes of the preterm infant from birth until discharge from the neonatal unit, including complications during hospitalization (e.g., respiratory distress, need for invasive ventilation, sepsis, feeding difficulties) and immediate post-discharge status within the first month at home. Long-term evolution referred to outcomes beyond the neonatal period, assessed up to the time of survey completion, and included developmental progress, growth, readmissions, ongoing need for medical care, and use of supportive therapies (e.g., physiotherapy, RSV prophylaxis). These definitions were applied consistently across data collection and analysis to ensure comparability between respondents. The evolution of preterm infants was assessed in two distinct periods: during hospitalization in the neonatal intensive care unit (NICU) and after discharge. Short-term evolution (hospitalization phase) was evaluated through questionnaire items addressing gestational age at birth, birth weight, Apgar scores, need for respiratory support (CPAP, intubation), duration of oxygen therapy, major complications (e.g., respiratory distress syndrome, sepsis, feeding difficulties), and discharge condition. Long-term evolution (post-discharge phase) was assessed through questions on enrollment in structured follow-up programs, frequency of medical reviews, readmissions, growth trajectory, achievement of developmental milestones, and use of supportive therapies such as physiotherapy and RSV prophylaxis. These self-reported data were supplemented by maternal recall of clinical diagnoses and interventions provided after discharge.

### 2.2. Study Population

The target population consisted of Romanian mothers aged ≥ 18 years who had at least one preterm live-born child. Preterm was defined as a birth that occurred before 37 weeks of pregnancy.

Inclusion Criteria:

- Romanian mothers aged ≥ 18 years.
- At least one preterm live-born child.
- Consent to participate and completion of the full questionnaire.

Exclusion Criteria:

- Incomplete questionnaire responses.

**Figure 1:**
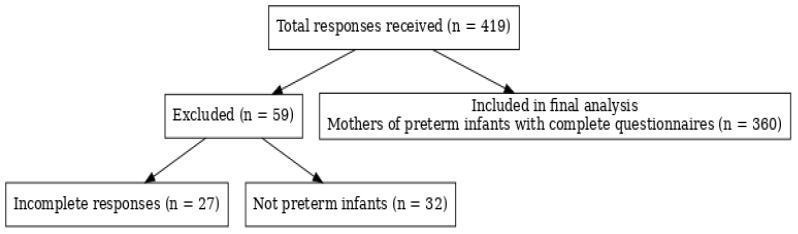
Study population selection.

A total of 419 responses were received. Of these, 27 were excluded due to incomplete questionnaires, and 32 were excluded because the respondent’s child was not born preterm. The final analysis included 360 mothers of preterm infants who completed the questionnaire in full (Figure 1).

### 2.3. Study Sample

The required sample size was estimated based on the primary outcome of interest, namely the proportion of preterm infants enrolled in a structured follow-up program after discharge. Assuming a prevalence of 25% for follow-up enrollment, a 95% confidence level (α = 0.05), and a precision (margin of error) of ±5%, the minimum sample size was calculated using the formula n = Z^2^ × p × (1 − p)/E^2^n = Z^2^ × p × (1 − p)/E^2^n = Z^2^ × p × (1 − p)/E^2^. This resulted in a requirement of 289 participants. The final study included 360 respondents, exceeding the minimum threshold and ensuring adequate statistical power for detecting differences of at least 15% between comparison groups at 80% power and α = 0.05.

### 2.4. Statistical Analysis

The data was then exported to Microsoft Excel 2013 and the statistical analyses were performed using IBM SPSS Statistics, Version 26, with a significance level set at *p* < 0.05. Continuous variables were tested for normality of distribution the Shapiro–Wilk test and the variance was tested with Levene’s test. The non-normally distributed variables were written as medians with interquartile ranges (IQRs). Quantitative variables were tested between independent groups using Mann–Whitney U tests. The Kruskal–Wallis test was used to determine significant differences between two or more groups of an independent variable. The normally distributed variables were written as mean with standard deviation (SD) and independent samples *t*-test was used to determine significant differences between groups. Fisher’s exact test was used to determine the nonrandom associations between categorical variables, with the Bonferroni method used for correction. A multiple linear regression was conducted to evaluate how well the method of conception and type of pregnancy predicted a mother’s age at birth, and the Durbin–Watson test indicated a reasonable level of independence in the errors (=1.82).

Given the cross-sectional, self-reported design we implemented several measures to reduce bias and to address confounding. The survey was anonymous to reduce social-desirability bias and encourage honest reporting. Data cleaning excluded incomplete questionnaires and excluded respondents whose infants were not preterm. Where possible, we used objective items (e.g., gestational age in weeks, type of delivery) rather than subjective ratings. For analyses we report bivariate comparisons and, when appropriate, multivariable models to adjust for potential confounders (e.g., maternal age, plurality, method of conception) when evaluating associations with outcomes such as gestational age or follow-up enrollment. Nonetheless, residual confounding and recall bias remain possible and are discussed as limitations: the cross-sectional design precludes causal inference.

### 2.5. Ethical Considerations

Informed consent was obtained from all subjects involved in the study. The study was approved by the Ethics Committee of the Clinical Hospital of Obstetrics and Gynecology “Prof. Dr. Panait Sîrbu”, Bucharest, Romania, under approval number 29/09.07.2025 and was conducted respecting the Helsinki Declaration for Human Rights.

## 3. Results

We had 360 answers. The majority (72.5%) of the answers came from mothers who gave birth in the 2020s decade. The median gestational age at birth was 32 (29–34) weeks, ranging from 23 to 36 weeks, and the majority (56.4%) were moderate to late preterm. The mean age of the mothers when they gave birth was 31.61 ± 5.3 years. A total of 72 (20%) of the pregnancies were obtained through artificial methods. Most of the births (80.8%) were through C-section. The majority, 277 (76.9%), were single pregnancies, and the rest, 83 (23.1%), were multiple pregnancies, with 78 (21.7%) twin pregnancy and 5 (1.4%) triplet pregnancy. The great majority of them (85.6%) gave birth in a level 3 maternity unit. A total of 74 (20.6%) patients were transferred from their maternity unit, 9 (12.2%) before birth and 65 (87.8%) after they gave birth. A total of 40 (11.1%) newborns had malformations at birth. Over half (53.6%) necessitated endotracheal (ET) intubation. The large majority of the newborns (86.1%) were admitted to the NICU. A summary of the respondents characteristics can be found in Table 2.

Maternal age at birth varied significantly across several maternal and perinatal factors (Table 3). Women who conceived through assisted reproductive techniques (ARTs) were, on average, 4.46 years older than those who conceived naturally (35.18 ± 5.67 vs. 30.72 ± 5.13 years; *p* < 0.001), although gestational age at birth did not differ significantly between these groups (*p* = 0.633). Cesarean delivery was associated with higher maternal age compared to vaginal delivery (32.02 ± 5.62 vs. 29.88 ± 4.82 years; mean difference = 2.14 years, 95% CI: 0.69 to 3.58; *p* = 0.004), while gestational age was lower in infants born vaginally (30 (26–33.5) vs. 32 (30–35) weeks; *p* < 0.001). Mothers with multiple pregnancies were older than those with singleton pregnancies (32.78 ± 5.38 vs. 31.26 ± 5.54 years; mean difference = 1.52 years, 95% CI: 0.16 to 2.87; *p* = 0.028), but gestational age was similar between groups (*p* = 0.297). Multiple pregnancies were significantly more frequent in the ART group compared to natural conception (48.6% vs. 16.7%; *p* < 0.001).

The rate of multiple pregnancy was much higher in those with artificial conception (48.6%) than in natural conception (16.7%) (*p* < 0.001). A linear regression was used to search for the factor associated with the maternal age between the method of conception and the type of pregnancy and resulted that, on average a mother who conceived through artificial means is 4.38 (95% CI: +1.61 to −1.09) years older than a mother who conceived naturally (*p* < 0.001); however, there is no meaningful difference in age between singleton pregnancy and multiple pregnancy once the method of conception is taken into account (*p* = 0.708).

There was no difference in the rate of malformations by mothers age (*p* = 0.371), gestational age at birth (*p* = 0.307), method of conception (*p* = 0.064), or singleton/multiple pregnancy (=0.842).

As it was expected, a lower gestational age was found in the ET intubation group, 30 (27–33) vs. 34 (32–35) years (*p* < 0.001).

**Figure 2:**
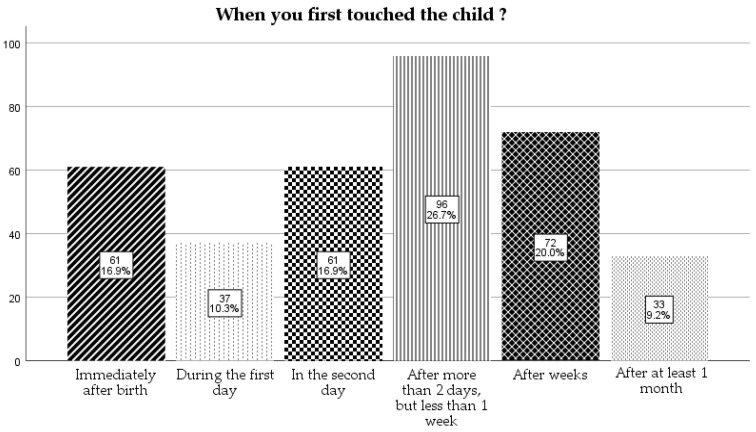
When the mother first touched their children.

We asked the mothers when they first touched the child and most of them responded that it was after more than 2 days, but less than a week (26.7%), followed by after weeks (20%). The detailed responses can be found in Figure 2.

**Figure 3:**
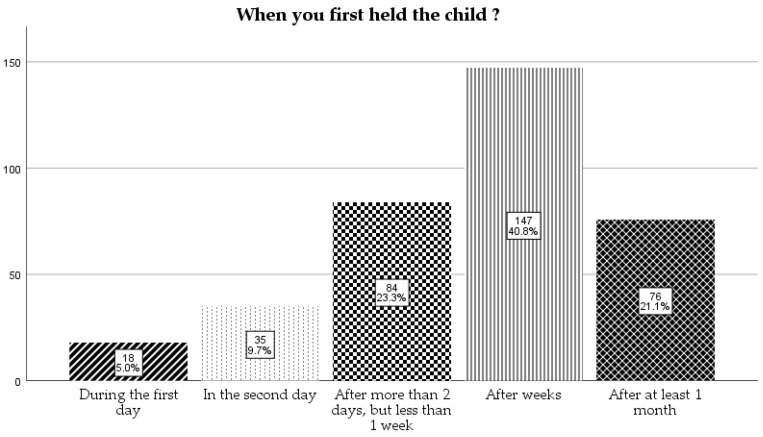
When the mother first held their children.

For the question “When you first held the child?”, most of them (41%) responded after 1 week, but less than 1 month, followed by on the 2nd day (23%) and after 1 month (21%) in similar proportions (Figure 3).

**Figure 4:**
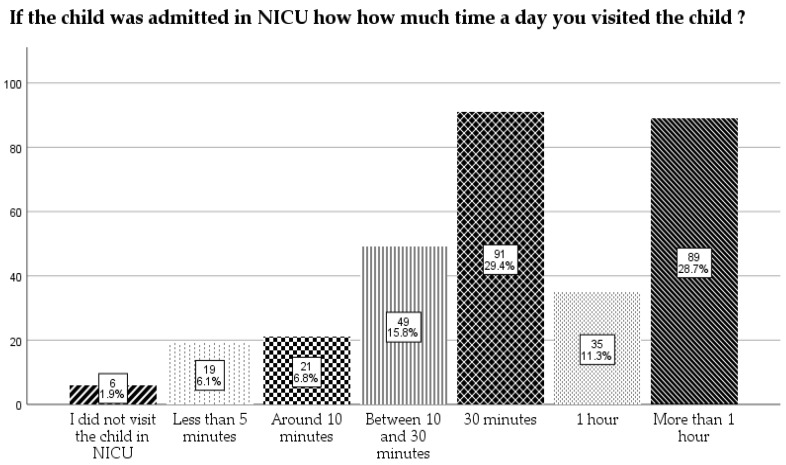
How much time a day did the mothers spend with the children in NICU.

The mothers were asked that if the child was admitted to NICU how much time a day they spent with the child. The most frequent answer was “around 30 min” (29.4%) followed by “more than 1 h” (28.7%), and the least of them were the ones that did not visit the child in NICU (1.9%) (Figure 4).

Only 59 (16.4%) of them responded that they had kangaroo mother care in maternity.

A little over half, 198 (55%), responded that the newborn was fed with breastmilk, pumped or directly, and 160 (80.8%) of them continued the breastfeeding at home. Of those 162 newborns who were not fed breastmilk in the admission period, 52 (32.1%) had changed the diet to breastfeeding at home. If we cumulate the numbers, it results that 212 (58.9%) had breastfed at home.

The large majority of the mothers (86.4%) responded that they were instructed by the medical staff about the need of further medical exams from other specializations.

**Figure 5:**
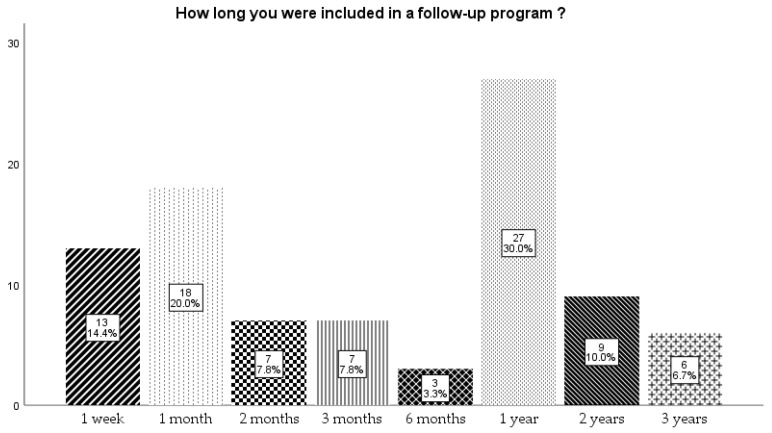
Time that newborns were included in a follow-up program.

Only a quarter (25%) of them had a follow-up program for the child at the maternity unit where they were born. The follow-up program varied from 1 week to 3 years, and most of them (30%) responded that they followed the program for 1 year (Figure 5). There was no association between mothers age at birth, area of provenance, education level, and the adherence to a follow-up program (*p* > 0.005).

We asked all the mothers (360) if they find the existence of the “Premature and Vulnerable Newborn’s Notebook” useful, and the vast majority (92.5%) responded “yes”.

Almost a half, 201 (55.8%), of them went with the child to physical therapy. Of those that went to physical therapy, three-quarters of them (76.1%) went to private (paid by themselves) and only 48 (23.9%) did physio in state institutions.

When asked if to their knowledge in their city there is a specialized center for monitoring premature babies, with all specializations, only one-third (31.7%) declared that they know about the existence of such a center.

Only 9.4% of them went to aquatic physiotherapy intervention.

When they were asked if they did the prevention of respiratory syncytial virus (RSV) infection with palivizumab, 148 (41.1%) responded that they did this prophylaxis for their preterm child.

**Figure 6:**
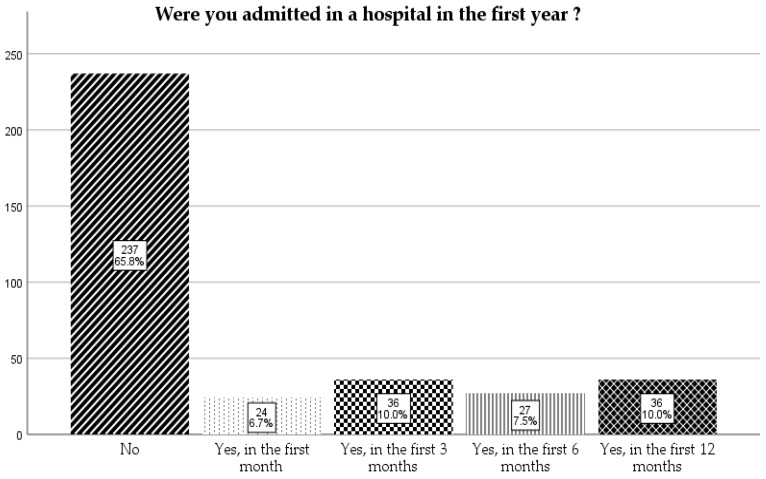
Admission to a hospital in the first year.

The majority of them (65.8%) were not admitted to a hospital in first year of life (Figure 6).

**Figure 7:**
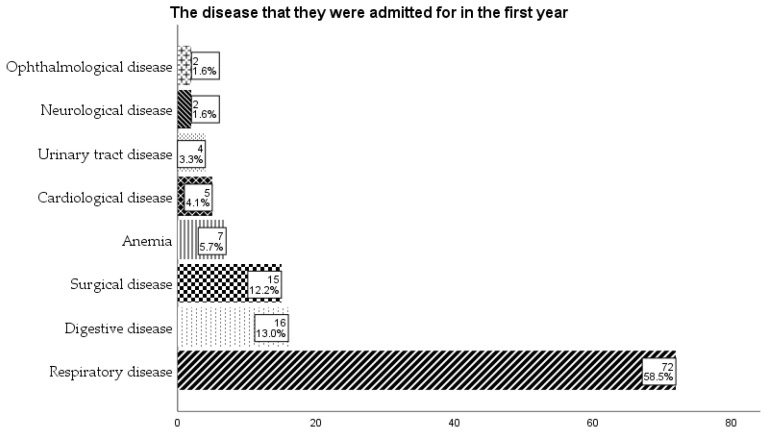
The diseases the children were admitted to the hospital in the first year.

Of those admitted in the first year, the majority (58.5%) had respiratory disease, followed by digestive disease (13%) and surgical disease (12.2%) (Figure 7).

Among the 360 preterm infants included, the most common short-term complications during hospitalization were respiratory distress (62.5%), need for CPAP (48.1%), and feeding difficulties requiring nasogastric feeding (45.6%). Endotracheal intubation was required in 18.9% of cases. The median length of NICU stay was 21 days (14–36). In the long term, 61.7% of infants were enrolled in a structured follow-up program, but only 38.4% continued beyond the first year of life. Readmissions within the first year were reported in 28.3% of cases, most commonly for respiratory infections. Physiotherapy was accessed by 41.1% of infants, RSV prophylaxis by 12.8%, and breastfeeding rates increased from 54.2% at discharge to 61.9% during follow-up. Developmental delays requiring specialist intervention were reported in 22.5% of children at the time of the survey.

## 4. Discussion

This study provides an overview of some of the perinatal characteristics and post-discharge follow-ups of premature infants in Romania. The results indicate a high rate of delivery in level 3 maternities, reflecting adherence to national protocols that promote centralization of high-risk births. However, 20.6% of cases required transfer from lower-level facilities, with the majority occurring postnatally, suggesting the need for better prenatal risk stratification and maternal transfer prior to delivery when feasible. Although a high proportion of births in our sample occurred in level 3 maternities, in line with national protocols, these results are specific to the study population and should not be generalized to all births in Romania without nationally representative data.

Maternal characteristics varied significantly by conception and pregnancy type. Mothers who conceived via assisted reproductive techniques were older and more likely to have multiple pregnancies. These findings align with national and international data and suggest that maternal age and infertility treatments remain important predictors of preterm birth and multiple gestation [31,32,33,34].

Although most infants were admitted to NICU and received respiratory support, including intubation, only a minority of mothers (16.4%) reported practicing kangaroo mother care, which is known to improve bonding, thermoregulation, growth, and breastfeeding rates [35,36,37]. The limited contact between mothers and infants in NICU as reported in this survey may reflect logistical constraints or cultural practices, and it represents an area for improvement in neonatal care policies.

Feeding practices remain a cornerstone of preterm infant outcomes. More than half of the infants received breastmilk during hospitalization, and this rate increased after discharge, with nearly 59% of infants being breastfed at home. These findings are encouraging, though they also underscore the need for enhanced lactation support in NICU settings to promote early and sustained breastfeeding [38,39].

In 2011, the Order of the Minister of Health No. 1232 of 2 August 2011 was published in the Official Gazette of Romania, Part I, No. 586 of 18 August 2011, specifying the need to follow-up premature babies until they reach 2 years of corrected age in level 3 maternity hospitals. However, there is no national registry of these premature babies, with each hospital having its own registry. In our maternity hospital, the follow-up of premature babies began in 2000 [40]. Despite recommendations for long-term monitoring of preterm infants, only 25% were enrolled in a structured follow-up program. This result is very concerning, with the follow-up rate described in other countries being 2 to 3 times higher, resulting a dire need of increasing this rate [41,42,43,44,45]. In the European Union, follow-up rates for children born very preterm vary widely between countries. Although one study reported that 90.3% of very preterm children received follow-up care initially, this proportion declined to 27.3% by the age of five years [46,47]. Nevertheless, the vast majority of mothers (92.5%) endorsed the utility of a “Premature and Vulnerable Newborn’s Notebook”, suggesting broad acceptability and the potential for high uptake if implementation is improved.

Our findings regarding limited parental contact in the NICU, low rates of kangaroo mother care, and variable awareness of specialized follow-up services are consistent with recent qualitative research on family perceptions of prematurity and early intervention. Silva et al. (2025) reported that parents of preterm infants often experience uncertainty about their child’s developmental prospects, perceive gaps in the continuity of care, and express a need for clearer guidance on neurodevelopmental follow-up and supportive services [9]. Such perceptions can influence parental engagement and adherence to early intervention programs, which are crucial for optimizing long-term neurodevelopmental outcomes. Addressing these informational and emotional needs through structured counseling, consistent communication between healthcare providers and families, and accessible educational resources could help improve both parental confidence and follow-up program participation rates in Romania.

Concerning access to multidisciplinary care, only 31.7% of mothers were aware of local specialized centers for preterm follow-up, and most physical therapy services were accessed privately. This highlights significant disparities in access to rehabilitation services and the underdevelopment of public healthcare infrastructure for the developmental surveillance of preterm infants.

Regarding health outcomes, 65.9% of infants were not readmitted in their first year of life, a reassuring finding. Among those who were, respiratory diseases dominated, followed by digestive and surgical conditions, a pattern consistent with international data on morbidity in preterm populations [14,15]. Notably, 41.1% of infants received RSV prophylaxis, showing relatively good uptake, especially considering that RSV preventive strategies were only recently expanded in Europe [48].

In this study, short-term evolution was primarily influenced by gestational age at birth. Lower gestational age was significantly associated with the need for endotracheal intubation and with vaginal delivery. Infants born vaginally had a median gestational age of 30 weeks compared to 32 weeks for those delivered by cesarean section (*p* < 0.001). Similarly, infants who required intubation had a median gestational age of 30 weeks compared to 34 weeks among those who did not require this intervention (*p* < 0.001). Long-term evolution defined as health status and medical interventions from discharge until the time of survey completion included access to supportive therapies (physiotherapy, RSV prophylaxis), continuation of breastfeeding, and the presence of a structured follow-up program. In our sample, availability of these services varied, which may influence the long-term prognosis of preterm infants. Notably, breastfeeding rates increased after discharge compared with during hospitalization, a finding that contrasts with the declining rates often reported in other studies.

### 4.1. Future Directions

One key initiative in development in Romania is the “Premature and Vulnerable Newborn’s Notebook”, designed to provide structured and accessible information to families regarding the necessary medical follow-up and developmental evaluations. Additionally, legislative efforts to extend parental leave to three years for Romanian mothers of preterm infants represent a recognition of the increased caregiving demands and long-term vulnerabilities faced by these children [28,29]. Despite these efforts, the most urgent need remains the implementation of a structured, national follow-up program for preterm infants, similar to those established in other high-income countries such as the Very Preterm Infant Register in Finland, the Swedish National Register for Retinopathy of Prematurity (SWEDROP), or the Korean Neonatal Network (KNN) [49,50,51].

### 4.2. Limitations

This study has several limitations that should be acknowledged. First, its cross-sectional design precludes causal inference; the associations observed cannot be interpreted as evidence of a direct influence on outcomes. Second, data were collected through a self-administered questionnaire, which is subject to recall bias, particularly for events occurring during neonatal hospitalization, and to social desirability bias, which may have led respondents to over-report favorable practices such as breastfeeding. Third, although the questionnaire underwent expert review to ensure face and content validity, it was not formally validated using psychometric methods, and clinical diagnoses were based on maternal reports rather than verification from medical records. Fourth, important prognostic variables such as birth weight, intrauterine growth status, and detailed neonatal morbidity data (e.g., bronchopulmonary dysplasia, intraventricular hemorrhage, periventricular leukomalacia, hearing impairment, retinopathy of prematurity) were not collected, limiting the ability to fully characterize risk profiles. Fifth, the study population included only mothers, excluding paternal perspectives that may provide complementary insights into parental awareness and engagement. Finally, participation was voluntary and recruitment was online, which may have introduced selection bias toward more motivated or resourceful parents; as such, the findings may not be fully representative of all families of preterm infants in Romania. Future research should address these limitations by using prospective designs with standardized clinical and developmental assessments, inclusion of both parents, and linkage to hospital records to enhance accuracy and generalizability.

## 5. Conclusions

This cross-sectional study sheds light on the multifactorial aspects influencing the evolution of preterm infants in Romania, both during hospitalization and after discharge. While the majority of births occurred in appropriately equipped centers and survival outcomes appear to be improving, there are notable gaps in post-discharge follow-up, parental support, and access to multidisciplinary care. The limited implementation of structured follow-up programs and reliance on private services for essential therapies highlight the need for stronger public healthcare infrastructure and improved resource allocation. Efforts such as the introduction of the “Premature and Vulnerable Newborn’s Notebook” and the expansion of RSV prophylaxis represent promising steps toward comprehensive care for preterm infants. However, more consistent national strategies and improved awareness among healthcare professionals and families are essential to ensure that every preterm infant receives equitable, high-quality care throughout their early development. The very low rate of only 25% involvement in follow-up is very concerning, and there is a dire need for interventions to increase this rate.

## Figures and Tables

**Table 1:** Structure and Content of the Study Questionnaire.

Section	Main Variables Collected	Example Questions
Maternal characteristics	Age at birth, education, residence, employment, method of conception	“What was your age when you gave birth?”
Pregnancy and delivery	Type of pregnancy, gestational age, delivery type, complications, maternity level, transfers	“Was the delivery by vaginal birth or C-section?”
Neonatal clinical data	Birth weight, congenital malformations, NICU admission, respiratory support	“Was your newborn admitted to the NICU?”
Parental contact in NICU	First touch/holding, daily visiting time, Kangaroo Mother Care	“On which day did you first hold your baby?”
Feeding practices	Feeding type in hospital, changes after discharge, breastfeeding duration	“Was your baby fed with breastmilk during hospitalization?”
Post-discharge follow-up	Enrollment in program, duration, awareness of specialized centers, specialist visits	“Was your baby included in a structured follow-up program?”
Supportive therapies	Physiotherapy, aquatic therapy, RSV prophylaxis	“Did your child receive RSV prophylaxis?”
Infant health outcomes	Readmissions in first year, causes of readmission	“Was your child admitted to hospital in the first year?”
Parental perceptions	Usefulness of “Premature and Vulnerable Newborn’s Notebook,” suggestions	“Do you consider the notebook useful?”

**Table 2:** Respondents characteristics.

Variable	N (%)	Median (IQR)/Mean (SD)
Age of the mother at birth (years)		31.61 ± 5.3
Gestational age at birth (weeks) Moderate to late preterm Very preterm Extremely preterm	203 (56.4%) 103 (28.6%) 54 (15%)	32 (29–34)
Decade of birth 2000s 2010s 2020s	18 (5%) 81 (22.5%) 261 (72.5%)	
Method of conception Natural Artificial	288 (80%) 72 (20%)	
Type of delivery Vaginal C-section	69 (19.2%) 291 (80.8%)	
Type of pregnancy Singleton Twin Triplets	277 (76.9%) 78 (21.7%) 5 (1.4%)	
Maternity level I II III	6 (1.7%) 46 (12.8%) 308 (85.6%)	
Malformations No Yes	320 (88.9%) 40 (11.1%)	
Endotracheal intubation No Yes	167 (46.4%) 193 (53.6%)	
Admission to NICU No Yes	50 (13.9%) 310 (86.1%)	
Type of pregnancy Singleton 277 (76.9%) Twin 78 (21.7%) Triplets 5 (1.4%) Maternity level I 6 (1.7%) II 46 (12.8%) III 308 (85.6%) Malformations No 320 (88.9%) Yes 40 (11.1%) Endotracheal intubation No 167 (46.4%) Yes 193 (53.6%) Admission to NICU Yes 310 (86.1%) No 50 (13.9%)		

**Table 3:** Maternal age and gestational age at birth by method of conception, type of delivery, and type of pregnancy.

Factor	Category	Maternal Age at Birth (Years)	Test Results (Maternal Age)	Gestational Age at Birth (Weeks)	Test Results (Gestational Age)	Interpretation
Method of conception	Natural	30.72 ± 5.13	F = 0.248, *p* > 0.05; Mean diff = −4.46 (95% CI: −5.82 to −3.10), *p* < 0.001	32 (29–34)	*p* = 0.633	ART mothers ~4.5 years older; GA similar
	Artificial	35.18 ± 5.67		32 (29–34)		
Type of delivery	Vaginal	29.88 ± 4.82	F = 0.113, *p* > 0.05; Mean diff = −2.14 (95% CI: −3.58 to −0.69), *p* = 0.004	30 (26–33.5)	*p* < 0.001	C-section mothers ~2 years older; vaginal births had lower GA
	Cesarean	32.02 ± 5.62		32 (30–35)		
Type of pregnancy	Singleton	31.26 ± 5.54	F = 0.158, *p* > 0.05; Mean diff = −1.52 (95% CI: −2.87 to −0.16), *p* = 0.028	32 (29–35)	*p* = 0.297	Multiple pregnancies had older mothers; GA similar
	Multiple	32.78 ± 5.38		32 (29–34)		

## Data Availability

Data are contained within the article.

## Abbreviations

The following abbreviations are used in this manuscript:

RSV	Respiratory Syncytial Virus
NICU	Neonatal Intensive Care Unit
BPD	Bronchopulmonary Dysplasia

## Appendix A

**Table A1:** Full questionnaire administered to mothers of preterm infants.

Section	Question Text	Response Format/Options
Maternal characteristics	What was your age when you gave birth to your preterm infant?	Numeric (years)
	What is your highest educational level?	Primary/Secondary/University/Postgraduate
	Place of residence	Urban/Rural
	Employment status at the time of birth	Employed/Unemployed/Student/Other
	How was the pregnancy obtained?	Natural conception/Assisted reproductive technology
Pregnancy and delivery details	Type of pregnancy	Singleton/Twin/Triplet
	Gestational age at delivery	Numeric (weeks)
	Type of delivery	Vaginal/C-section
	Did you have any pregnancy complications?	Yes/No (If yes, specify)
	In which level of maternity hospital did you deliver?	Level I/Level II/Level III
	Were you transferred from another hospital before or after delivery?	Yes, before delivery/Yes, after delivery/No
Neonatal clinical data	Birth weight	Numeric (grams)
	Did your newborn have congenital malformations?	Yes/No
	Was your newborn admitted to the NICU?	Yes/No
	Did your newborn require endotracheal intubation?	Yes/No
Parental contact in NICU	On which day after birth did you first touch your baby?	Numeric (days)
	On which day after birth did you first hold your baby?	Numeric (days)
	How much time per day did you usually spend with your baby in the NICU?	Did not visit/Less than 30 min/Around 30 min/30 min–1 h/More than 1 h
	Did you practice skin-to-skin contact (Kangaroo Mother Care) during hospitalization?	Yes/No
Feeding practices	What type of feeding did your baby receive during hospitalization?	Breastmilk (direct or expressed)/Formula/Mixed
	Did feeding change after discharge?	Yes/No (If yes, specify: to breastmilk/to formula/to mixed)
Post-discharge follow-up	Was your baby included in a structured follow-up program at the maternity where they were born	Yes/No
	If yes, how long did the follow-up program last?	Less than 1 week/1 week–1 month/1–3 months/3–6 months/6 months–1 year/More than 1 year
	Were you instructed by medical staff about the need for further specialist medical exams?	Yes/No
	Are you aware of a specialized center for monitoring premature babies in your city?	Yes/No
Access to supportive therapies	Did your child receive physical therapy?	Yes/No
	If yes, where?	State institution/Private clinic
	Did your child receive aquatic therapy?	Yes/No
	Did your child receive RSV prophylaxis with Palivizumab?	Yes/No
Infant health outcomes in first year	Was your child admitted to hospital in the first year of life?	Yes/No
	If yes, what was the main cause?	Respiratory disease/Digestive disease/Surgical disease/Other (specify)
	Did you administer Palivizumab for the prevention of respiratory RSV infection ?	Yes/No
Parental perceptions and proposals	Do you consider the “Premature and Vulnerable Newborn’s Notebook” useful?	Yes/No
	Do you have any additional comments or suggestions regarding the care of premature infants?	Open-ended
